# Linear accelerator–based stereotactic arrhythmia radioablation for paroxysmal atrial fibrillation in elderly: a prospective phase II trial

**DOI:** 10.1093/europace/euad344

**Published:** 2023-11-21

**Authors:** Antonio Di Monaco, Fabiana Gregucci, Ilaria Bonaparte, Imma Romanazzi, Federica Troisi, Alessia Surgo, Nicola Vitulano, Federico Quadrini, Noemi Valenti, Roberta Carbonara, Fiorella Cristina Di Guglielmo, Elena Ludovico, Roberto Calbi, Pietro Guida, Maria Paola Ciliberti, Alba Fiorentino, Massimo Grimaldi

**Affiliations:** Department of Cardiology, General Regional Hospital ‘F. Miulli’, Acquaviva delle Fonti 70021, Bari, Italy; Department of Clinical and Experimental Medicine, University of Foggia, Viale Luigi Pinto 71122 Foggia, Italy; Department of Radiation Oncology, General Regional Hospital ‘F. Miulli’, Acquaviva delle Fonti, Bari, Italy; Department of Radiation Oncology, General Regional Hospital ‘F. Miulli’, Acquaviva delle Fonti, Bari, Italy; Department of Cardiology, General Regional Hospital ‘F. Miulli’, Acquaviva delle Fonti 70021, Bari, Italy; Department of Cardiology, General Regional Hospital ‘F. Miulli’, Acquaviva delle Fonti 70021, Bari, Italy; Department of Radiation Oncology, General Regional Hospital ‘F. Miulli’, Acquaviva delle Fonti, Bari, Italy; Department of Cardiology, General Regional Hospital ‘F. Miulli’, Acquaviva delle Fonti 70021, Bari, Italy; Department of Cardiology, General Regional Hospital ‘F. Miulli’, Acquaviva delle Fonti 70021, Bari, Italy; Department of Cardiology, General Regional Hospital ‘F. Miulli’, Acquaviva delle Fonti 70021, Bari, Italy; Department of Radiation Oncology, General Regional Hospital ‘F. Miulli’, Acquaviva delle Fonti, Bari, Italy; Department of Radiation Oncology, General Regional Hospital ‘F. Miulli’, Acquaviva delle Fonti, Bari, Italy; Department of Radiology, General Regional Hospital ‘F. Miulli’, Acquaviva delle Fonti, Bari, Italy; Department of Radiology, General Regional Hospital ‘F. Miulli’, Acquaviva delle Fonti, Bari, Italy; Department of Cardiology, General Regional Hospital ‘F. Miulli’, Acquaviva delle Fonti 70021, Bari, Italy; Department of Radiation Oncology, General Regional Hospital ‘F. Miulli’, Acquaviva delle Fonti, Bari, Italy; Department of Radiation Oncology, General Regional Hospital ‘F. Miulli’, Acquaviva delle Fonti, Bari, Italy; Department of Medicine and Surgery, LUM University, Casamassima, Bari, Italy; Department of Cardiology, General Regional Hospital ‘F. Miulli’, Acquaviva delle Fonti 70021, Bari, Italy

**Keywords:** Atrial fibrillation, Stereotactic arrhythmia radioablation, Elderly, Pulmonary vein isolation

## Abstract

**Aims:**

Stereotactic arrhythmia radioablation (STAR) is a novel therapeutic approach for cardiac arrhythmias. The aim of this trial is to investigate the feasibility of STAR for the treatment of paroxysmal atrial fibrillation (AF) in elderly patients.

**Methods and results:**

Inclusion criteria were age >70 years, symptomatic AF, antiarrhythmic drugs failure, or intolerance. All patients underwent to 4D cardiac computed tomography simulation. The clinical target volume was identified in the area around pulmonary veins (PV). Stereotactic arrhythmia radioablation was performed with a total dose of 25 Gy (single fraction) delivered in 3 min. Twenty patients were enrolled and 18 underwent STAR. One patient withdrew informed consent before treatment and one patient was excluded due to unfavourable oesophagus position. With a median follow-up (FU) of 16 months (range 12–23), no acute toxicity more than Grade 3 was reported. Five patients had a Grade 1 oesophagitis 24 h after STAR; eight patients had an asymptomatic Grade 1 pericardial effusion, and one patient had a torsade de pointes treated effectively by electrical cardioversion and subsequent cardiac implantable cardioverter-defibrillator implantation. Most patients had a significant reduction in AF episodes. Five patients, due to arrhythmias recurrences after STAR, performed electrophysiological study documenting successful PV isolation. Finally, a significant improvement of quality of life was documented (48 ± 15 at enrolment vs. 75 ± 15 at 12 months FU; *P* < 0.001).

**Conclusion:**

The present phase II trial demonstrated the feasibility of STAR in paroxysmal AF elderly patients and its potential role in increasing the quality of life. Surely, more robust data are needed about safety and efficacy.

**Trial registration:**

ClinicalTrials.gov: NCT04575662

## Graphical Abstract

**Graphical Abstract:**
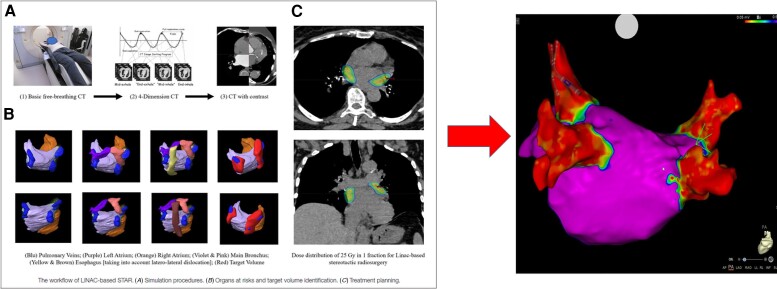

**What’s new?**

- Stereotactic arrhythmia radioablation (STAR) is a novel non-invasive therapeutic approach for cardiac arrhythmias.
- The present phase II trial demonstrated the feasibility of STAR in elderly patients affected by paroxysmal atrial fibrillation, reporting also promising data in terms of safety, outcomes, and quality of life.
- Stereotactic arrhythmia radioablation treatment was able to perform pulmonary vein isolation as invasive catheter ablation.
- This new non-invasive therapeutic approach can represent a valid alternative to treat paroxysmal atrial fibrillation in elderly.

## Introduction

Stereotactic arrhythmia radioablation (STAR) is a novel therapeutic approach for cardiac arrhythmia.^1,2^ Stereotactic arrhythmia radioablation reported a great and multifactorial antiarrhythmic effect whose mechanism of action is unknown.^2–4^ Several STAR data were published for ventricular tachycardia (VT), using different technologies, including linear accelerators (LINACs) or Cyberknife, reporting a reduction of VT episodes after treatment.^1,2,4–9^

For atrial fibrillation (AF), the data are sparse.^1,3–5,10–14^ Atrial fibrillation is the most common cardiac arrhythmia in elderly, and due to the population ageing, its prevalence is growing up.^15,16^ In the European Union in 2060, the number of patients older than 75 years with AF is estimated to be 13.8 million.^17^ In this context, paroxysmal AF is difficult to treat with drugs, due to the frequent AV node conduction or intraventricular conduction delays or due to tachy-brady syndrome. Furthermore, ablation of the atrioventricular node and pacemaker implantation can control ventricular rate when medication fails.^15,18^

Current guidelines recommend catheter ablation targeting pulmonary vein isolation (PVI) to treat paroxysmal AF refractory to antiarrhythmic therapy (AAT).^15,19,20^ Nevertheless, catheter ablation is burdened with an increased risk of complication rate in elderly.^15,21,22^

This prospective phase II trial was designed to evaluate safety of STAR in elderly patients affected by paroxysmal AF (ClinicalTrials.gov: NCT04575662).

## Material and methods

The trial was approved by the Ethics Committee and all patients signed informed consent. Inclusion criteria were age >70 years, symptomatic paroxysmal AF, and AAT intolerance or failure. Exclusion criteria were persistent or permanent AF, previous AF ablation, unstable angina, life expectancy to <1 year, previous cardiac surgery, myocardial infarction or thromboembolic events, contraindications to oral anticoagulation, and active systemic infection. After enrolment, all patients discontinued AAT and they performed 15-day electrocardiogram (ECG)-Holter monitoring, a complete transthoracic echocardiogram, and a cardiac computed tomography (CT) before STAR. The EuroQol (EQ) visual analogue scale (VAS) was used to assess quality of life, administrated before treatment and 12 months after it. The EQ VAS records the patient’s self-rated health on a vertical VAS from 0 (the worst health you can imagine) to 100 (the best health you can imagine).^23^

### Radiotherapy

Stereotactic arrhythmia radioablation procedure was recently published: patients underwent to simulation with a 4D CT, with an immobilization system, and received a free breathing STAR with a prescription total dose of 25 Gy in 1 fraction. A ‘simultaneous integrated protection’ dose was realized to the interface between PVs and critical structures, including oesophagus and bronchus, to respect dose constraint value.^13,24–26^ The treatment was generated, optimized, and delivered by TrueBeam^TM^ (Varian Medical System, Palo Alto, CA). Image-guided radiotherapy (IGRT) with cone beam CT and surface-guided radiotherapy (SGRT) with AlignRT (Vision RT) were used to reduce set-up error and to monitor patients during fraction. Radiotherapy delivery was temporally interrupted in case of deep breaths.

### Clinical follow-up

All patients were clinically monitored during procedure and for 12 h after STAR. Follow-up (FU) consisted of clinical evaluation, 15-day ECG-Holter monitoring, and transthoracic echocardiogram performed at 1, 3, 6, and 12 months after STAR. Cardiac CT was performed at 6 months from STAR.

### Study objectives

The primary endpoint was to assess the 1-month post-STAR safety defined as followed: complete STAR delivery and no acute treatment-related adverse events more than Grade 3, assessed according to the Common Terminology Criteria for Adverse Events (version 5.0).^27^ Secondary endpoints were AF recurrences (episodes ≥30 s) carried out through ECG or ECG-Holter monitoring, reductions in AAT, quality of life, and overall survival.

### Sample size and statistical analysis

Based on the exploratory nature of study, the planned number of patients was 20 without a formal statistical power calculation. Summary statistics were reported as number of subjects, frequency with percentage for categorical data, and median or mean ± standard deviations for continuous variables. The incidence rate ratios (IRR) with 95% confidence interval for the number of AF episodes recorded during ECG-Holter monitoring (pre-treatment and 1, 3, 6, and 12 months post-treatment) were calculated using a Poisson regression mixed model with patient as random effect. A *P* value of 0.05 or less was considered statistically significant. Wilcoxon matched-pairs signed-rank test was used to compare quality of life 12 months post-treatment than pre-treatment. All analyses were performed using STATA version 16 (StataCorp, College Station, Texas).

## Results

**Figure 1:**
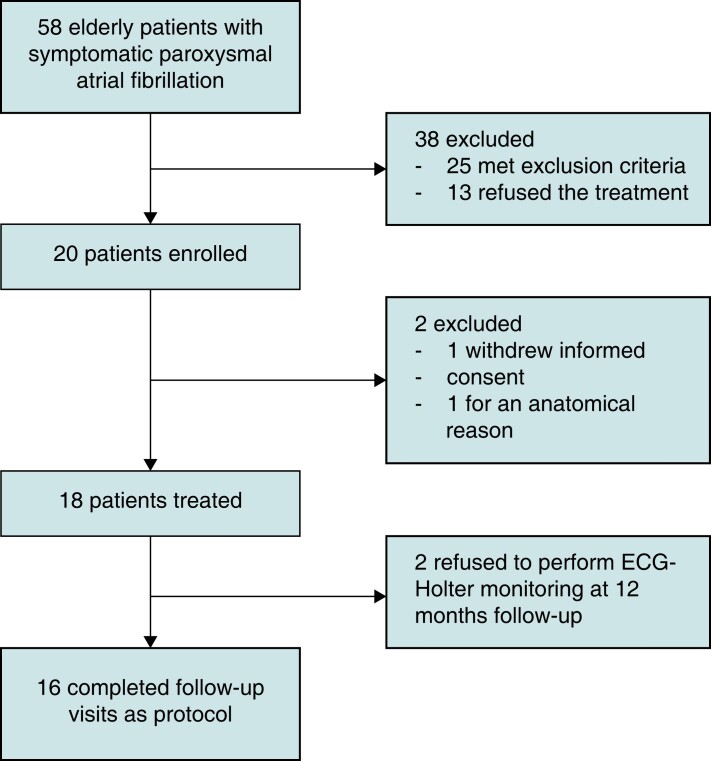
Flow diagram regarding the STAR clinical trial.

From May 2021 to July 2022, 20 patients were enrolled on 20 planned (100%). Eighteen patients underwent STAR. One patient withdrew informed consent before treatment, and one patient was excluded due to the close relationship between the oesophagus and the left PVs (high risk of oesophageal toxicity or high risk to perform a non-effective treatment) (*Figure 1*). The main clinical characteristics of patients were reported in *Table 1*.

**Table 1:** Clinical characteristics of the 20 study patients

Male sex, *n* (%)	8 (40)
Mean age (years)	77 ± 6
CHA_2_DS_2_-VASc, *n* (%)	
0	0 (0)
1	1 (5)
2	1 (5)
3	12 (60)
4	6 (30)
EHRA score, *n* (%)	
2 A	—
2 B	—
3	16 (80)
4	4 (20)
Diabetes mellitus, *n* (%)	1 (5)
Hypertension, *n* (%)	16 (80)
Family history of coronary artery disease, *n* (%)	15 (75)
Hypercholesterolaemia, *n* (%)	11 (55)
Hypertriglyceridaemia, *n* (%)	9 (45)
Active smoking, *n* (%)	6 (30)
Body mass index (kg/m^2)^	26 ± 3
Heart failure (EF < 35%), *n* (%)	0 (0)
Coronary artery disease, *n* (%)	1 (5)
Previous ischaemic stroke, *n* (%)	1 (5)
Transient ischaemic attack, *n* (%)	1 (5)
Chronic renal failure, *n* (%)	7 (33)
Dysthyroidysm, *n* (%)	7 (35)
Chronic lung disease, *n* (%)	4 (20)
Medical therapy, *n* (%)	
Beta-blockers, *n* (%)	15 (75)
Flecainide, *n* (%)	7 (35)
Propafenon, *n* (%)	1 (5)
Amiodaron, *n* (%)	5 (25)
Sotalol, *n* (%)	2 (10)
Direct oral anticoagulant, *n* (%)	20 (100)

EF, ejection fraction; EHRA, European Heart Rhythm Association.

**Figure 2:**
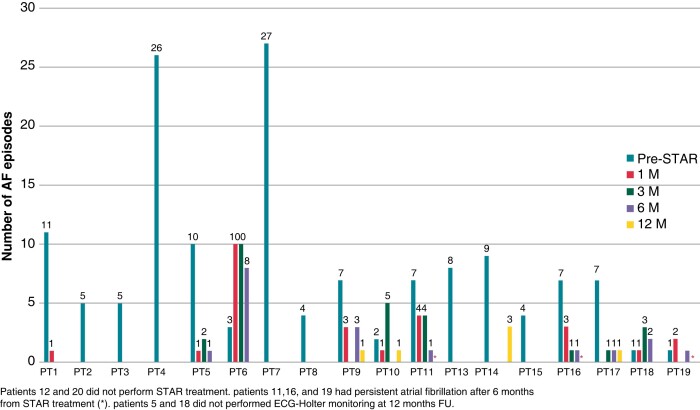
Atrial fibrillation episodes documented during 15-day ECG-Holter monitoring.

Before treatment, all patients performed 15-day ECG-Holter monitoring, reporting a median of eight AF episodes (range 1–27) (*Figure 2*). Furthermore, frequent premature atrial beats (>1000/24 h) without episodes of bradycardia or ventricular arrhythmias were recorded. Data regarding the T0 echocardiography were reported in *Table 2*. No significant findings were documented at the baseline chest/cardiac CT.

**Table 2:** Baseline echocardiographic data (20 patients)

Left ventricle ejection fraction (%)	55 ± 5
Left ventricle hypertrophy (septum diameter >12 mm), *n* (%)	13 (65)
Right ventricle dysfunction, *n* (%) (defined as TAPSE < 16 mm and S′ <10 cm/s)	—
Mean left atrial anterior–posterior diameter (mm)	44 ± 5
Mean left atrial area (cm^2^)	22 ± 4
Right atrium enlargement, *n* (%) (2D right atrial volume >30 mL/m^2^)	5 (25)
Mitral valve regurgitation, *n* (%)	
Mild	11 (55)
Moderate	3 (15)
Moderate-to-severe	1 (5)
Severe	—
Mitral valve stenosis, *n* (%)	—
Aortic valve regurgitation, *n* (%)	
Mild	5 (25)
Moderate	1 (5)
Moderate-to-severe	—
Severe	—
Aortic valve stenosis, *n* (%)	—
Tricuspid valve regurgitation, *n* (%)	
Mild	12 (60)
Moderate	3 (15)
Moderate-to-severe	1 (5)
Severe	—
Tricuspid valve stenosis, *n* (%)	—
Pericardial effusion, *n* (%)	—

TAPSE, tricuspid annular plane systolic excursion.

In terms of RT data, regarding dose constraints, average heart and left anterior descending artery (LAD) mean dose was 3.9 and 6.3 Gy, respectively, while mean maximum dose for LAD, spinal cord, left and right bronchus, and oesophagus was 11.2, 7.5, 14.3, 12.4, and 13.6 Gy, respectively.

From geometrical point of view, STAR plan was delivered using three non-coplanar arcs with 10 MV-flattening filter free (FFF). The median delivering treatment time was 3 min. All patients received a total dose of 25 Gy in a single fraction on PVs.

### Primary endpoint

With a median FU time of 16 months (range 12–23), no acute toxicity more than Grade 3 surely related with irradiation was reported. All adverse events were reported in *Table 3*.

**Table 3:** Adverse event classification

	Grade 1				Grade 2			
	Unrelated	Unlikely	Possible	Probable	Unrelated	Unlikely	Possible	Probable
Cardiac disorder								
Sustained VT								
Pericarditis				8				
Coronary embolism								
AV block								
GI disorder								
Oesophagitis				6				

	Grade 3				Grade 4			
	Unrelated	Unlikely	Possible	Probable	Unrelated	Unlikely	Possible	Probable
Cardiac disorder								
Sustained VT							1	
Pericarditis			1					
Coronary embolism					1			
AV block					1			
GI disorder								
Oesophagitis								

AV, atrioventricular; GI, gastrointestinal; VT, ventricular tachycardia.

Five patients (27.7%) had a mild oesophagitis (Grade 1) 24 h after STAR; the symptoms resolved after 1 week using proton pump inhibitors and sucralfate. Five months after STAR, 1 patient (5.5%) had epigastric pain unresponsive to pharmacological treatment using proton inhibitors and sucralfate. The patient underwent chest CT and endoscopy not documenting oesophageal damage. After discharge, the patient developed herpes zoster infection at the site of pain symptoms.

Furthermore, eight patients (44.5%) experienced an asymptomatic mild (Grade 1) pericardial effusion (max 2 mm): 2 patients after 1 month from STAR, and their pericardial effusion completely resolved in 3 months; 6 patients after 6 months from STAR, and 1 out 6 had a complete resolution, while the other 5 patients had a stable asymptomatic mild pericardial effusion at 1 year FU.

Only 1 patient (5.5%) had a symptomatic (Grade 3) pericardial effusion (about 5 mm) documented after 6 months from STAR; the pericardial effusion completely resolved in 2 months using pharmacological treatment with corticosteroids. No other significant alterations were found at echocardiograms performed during the FU visits.

One patient had a clinically significant acute event after STAR: after 1 h from treatment Patient 13 had a torsade de pointes treated effectively by electrical cardioversion and subsequent cardiac implantable cardioverter-defibrillator (ICD) implantation.

Two adverse events were not related to STAR. In particular, one patient had a coronary artery embolism after electrical cardioversion for AF performed 4 months after STAR. The patient did not perform exams to exclude atrial thrombus because anticoagulant therapy had been taken, without interruption, for more than 3 weeks before electrical cardioversion. Moreover, 1 patient had a dual-chamber pacemaker implanted 13 months after STAR for a sinus node disease progression. An asymptomatic mild sinus node dysfunction was documented before enrolment for which beta-blocker therapy was discontinued.

No significant alterations were documented on the chest CT scan performed 6 months after STAR.

### Secondary endpoint

Frequent atrial ectopies (>1000/24 h) and atrial tachycardias episodes were documented in all patients during the first 2 months after STAR. Most patients had a significant reduction in AF episodes during FU (*Figure 2*). Compared with pre-treatment number of AF episodes (eight per patient over 15 days), the risk was significantly lower at each post-baseline time point with IRRs indicating a reduction of >80% (1, 3, 6, and 12 months) (*Table 4*). The median value of maximal AF duration was 10 h (range 1–36). Seven patients were arrhythmia-free during FU (*Figure 2*). Three patients (16.6%) developed persistent AF after 6 months from STAR (Patients 11, 16 and 19). In particular, Patient 11 had a pericardial effusion, and Patients 16 and 19 had a significant valve disease (moderate-to-severe mitral and tricuspid regurgitation).

**Table 4:** Incidence rate of atrial fibrillation episodes by time since STAR treatment and risk of atrial fibrillation for time at risk after thereafter

	Events per 15 days	Incidence rate ratios	*P*
Pre-treatment	8.00 (6.80–9.42)	1.00	
1 month	1.44 (0.98–2.12)	0.18 (0.12–0.27)	<0.001
3 months	1.44 (0.98–2.12)	0.18 (0.12–0.27)	<0.001
6 months	1.00 (0.63–1.59)	0.13 (0.08–0.20)	<0.001
12 months	0.56 (0.29–1.08)	0.07 (0.04–0.14)	<0.001

Patients 1 and 3 had a symptomatic atypical atrial flutter at 6 months post-STAR, while Patient 13 had an episode of atypical atrial flutter after 12 months. No other significant arrhythmias were documented.

All the patients with arrhythmia recurrences resumed AAT with beta-blockers; one patient started flecainide 6 months from STAR, and two patients started amiodarone after 3 and 12 months from STAR.

All patients with recurrent arrhythmias were offered to undergo an electrophysiological study (ES), but most of them refused due to a significant improvement in symptoms. Five patients performed ES (Patients 1, 3, and 13 presenting an atypical atrial flutter and Patients 5 and 9 with paroxysmal AF). In these patients, the atrial mapping using CARTO System and Pentaray or Octaray mapping catheters (Biosense Webster, CA, USA) were performed and no PV stenosis or phrenic nerve damage was documented.

**Figure 3:**
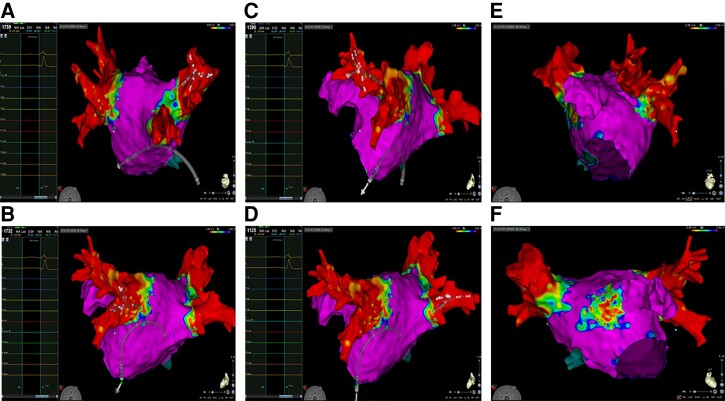
Electroanatomic mapping of the left atrium using CARTO System and Pentaray catheter. (*A*) Pentaray catheter inserted into the right superior pulmonary vein documenting the absence of electrical potentials. (*B*) Pentaray catheter inserted into the left inferior pulmonary vein documenting the absence of electrical potentials. (*C*) Pentaray catheter inserted into the left superior pulmonary vein documenting the absence of electrical potentials. (*D*) Pentaray catheter inserted into the right inferior pulmonary vein documenting the absence of electrical potentials. (*E*) Left lateral view of the left atrium. (*F*) Anterior view documenting a low-voltage area on the roof of the left atrium. This area was the site of atrial tachycardia.

**Figure 4:**
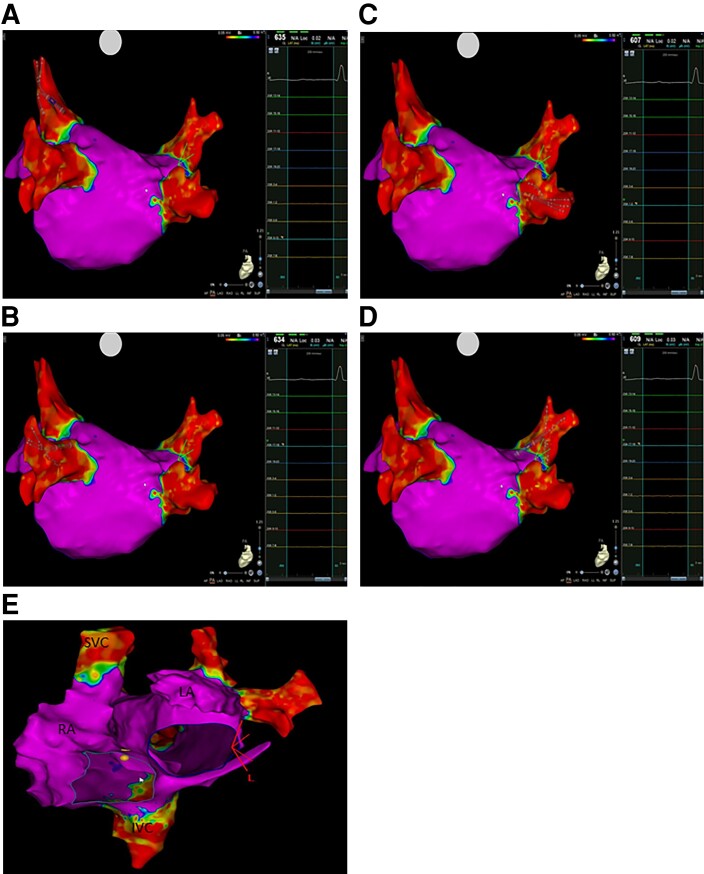
Electroanatomic mapping of the left atrium using CARTO System and Pentaray catheter. (*A*) Pentaray catheter inserted into the left superior pulmonary vein documenting the absence of electrical potentials. (*B*) Pentaray catheter inserted into the left inferior pulmonary vein documenting the absence of electrical potentials. (*C*) Pentaray catheter inserted into the right inferior pulmonary vein documenting the absence of electrical potentials. (*D*) Pentaray catheter inserted into the right superior pulmonary vein documenting the absence of electrical potentials. (*E*) Right and left atrial electroanatomic mapping.

Electrophysiological study performed after 6 months from STAR:

- Patient 1: PVI was documented; high-rate atrial stimulation induced the clinical atrial flutter whose critical isthmus was ablated in a low-voltage area in the anterior wall (this area was far from targeted areas of STAR) (*Figure 3*).
- Patient 3: ES documented PVI, absence of low-voltage areas in both atria. Programmed and high-rate atrial stimulation was not able to induce any arrhythmia (*Figure 4*).

**Figure 5:**
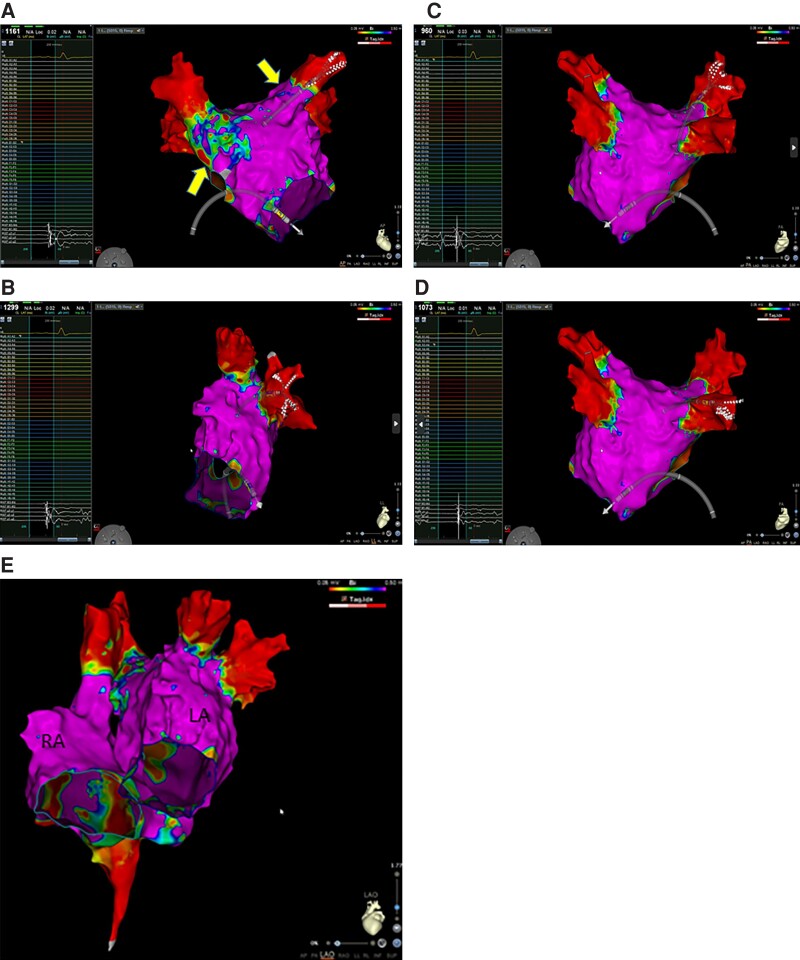
Electroanatomic mapping of the left and right atrium using CARTO System and Octaray catheter. (*A*) Octaray catheter inserted into the left superior pulmonary vein documenting the absence of electrical potentials. Arrows represent the areas close to oesophagus and left bronchus. (*B*) Octaray catheter inserted into the left inferior pulmonary vein documenting the absence of electrical potentials. (*C*) Octaray catheter inserted into the right superior pulmonary vein documenting the absence of electrical potentials. (*D*) Octaray catheter inserted into the right inferior pulmonary vein documenting the absence of electrical potentials. (*E*) Right and left atrial electroanatomic mapping.

**Figure 6:**
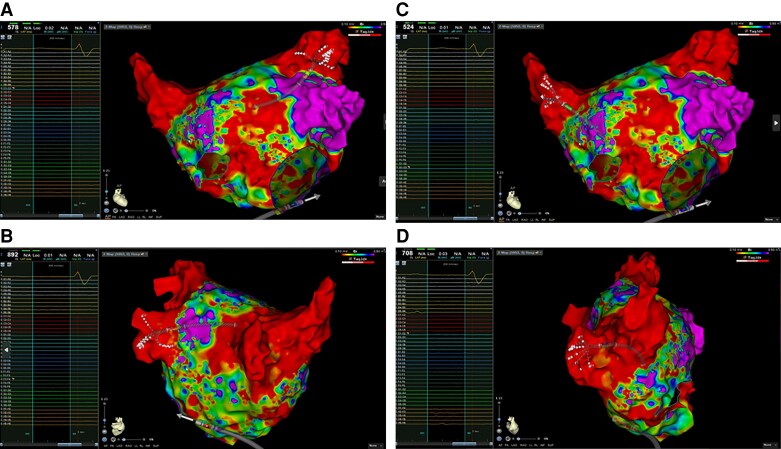
Electroanatomic mapping of the left atrium using CARTO System and Octaray catheter. (*A*) Octaray catheter inserted into the left superior pulmonary vein documenting the absence of electrical potentials. (*B*) Octaray catheter inserted into the left inferior pulmonary vein documenting the absence of electrical potentials. (*C*) Octaray catheter inserted into the right superior pulmonary vein documenting the absence of electrical potentials. (*D*) Octaray catheter inserted into the right inferior pulmonary vein documenting the absence of electrical potentials.

**Figure 7:**
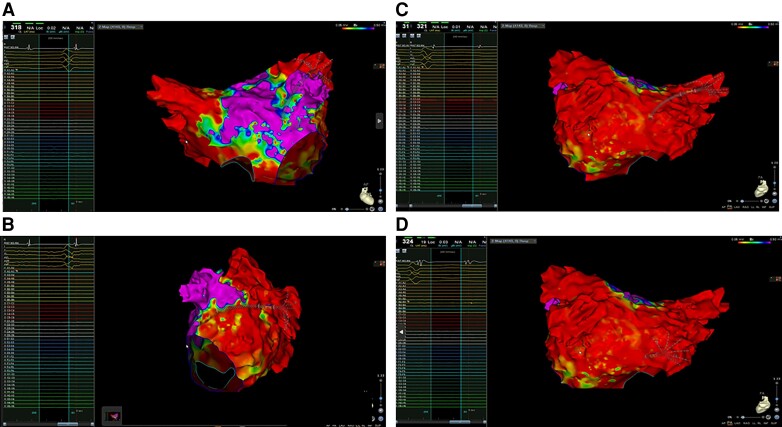
Electroanatomic mapping of the left atrium using CARTO System and Pentaray catheter. (*A*) Octaray catheter inserted into the left superior pulmonary vein documenting the absence of electrical potentials. (*B*) Octaray catheter inserted into the left inferior pulmonary vein documenting the absence of electrical potentials. (*C*) Octaray catheter inserted into the right superior pulmonary vein documenting the absence of electrical potentials. (*D*) Octaray catheter inserted into the right inferior pulmonary vein documenting the absence of electrical potentials.

Electrophysiological study performed after 12 months from STAR:

- Patient 9 documented PVI with lesions performed distally in the superior veins (*Figure 5*). In this patient, the oesophagus and left bronchus were located close to the superior PV ostia and the STAR was planned deeper in the veins to avoid their damage. In this patient, antral electrical activity on the superior PVs was ablated.
- Patient 5 had PVI with high grade of left atrial fibrosis (*Figure 6*). No rotational or focal activity was detected by CARTO Finder software (Biosense Webster, CA, USA); posterior wall isolation was performed.
- Patient 13 had PVI, and left atrial posterior wall did not show any electrical activity (*Figure 7*); critical isthmus of left atrial flutter was ablated on the left atrial roof.

A significant improvement of quality of life was documented after STAR (48 ± 15 at enrolment vs. 75 ± 15 at 12 months FU; *P* < 0.001). No patient died.

## Discussion

Atrial fibrillation is the most common cardiac arrhythmia with an increased risk of complications especially if a proper anticoagulation or a proper rhythm and rate control is lacking.^15–22^

Pulmonary vein isolation remains the cornerstone of AF ablations.^19^ In elderly, this procedure is often not performed due to the higher risk of procedural complications: vascular injury, cardiac perforation, phrenic nerve injury, stroke, and most concerning, atrio-oesophageal fistula, with high mortality rate.^19–22^ For these reasons, in the clinical practice, it is preferred to use pharmacological treatment rather than interventional procedures. However, AAT can lead to serious side effects or it is contraindicated due to sinus bradycardia or conduction disorders.^15^ The ablation of the atrioventricular node and pacemaker implantation can control ventricular rate when medication fails, however exposing the patient to the risk of device malfunction and infection.^15,18^ Moreover, many of patients enrolled in this study refused interventional therapies.

As a result, there is ongoing interest in developing improved therapies for AF and non-invasive therapeutic alternatives are warranted. The STAR study was born from the desire to obtain PVI using a non-invasive method, suitable for older patients or those who cannot have or not desire an invasive procedure. The outpatient care setting allows for the treatment of a large number of patients without occupying hospital beds. This aspect is of great importance considering the large number of patients eligible for the procedure as opposed to the progressive reduction of hospital beds.

The present phase II LINAC-STAR trial is the first worldwide study designed to evaluate the safety of this treatment. In terms of safety endpoint, the adverse events with a possible correlation to STAR were mild oesophagitis and mild pericardial effusion among which only one required pharmacological therapy.

Oesophagitis is essentially due to the anatomical proximity of the oesophagus and the PVs, and we documented mild oesophagitis without the onset of fistulas in a longer follow-up. We performed a ‘simultaneous integrated protection’ dose for the oesophagus and bronchus, for reducing the risk of side effect. Moreover, STAR was delivered using IGRT and SGRT to increase precision of target position and organs at risk and the delivering treatment time was very short (3 min instead of 45–90 min for other technologies), reducing the risk of oesophagus displacement during a longer treatment.^12–14^ Prophylactic treatment to prevent oesophagitis should be considered in the future.

The patient’s anatomy is essential for structuring an optimal treatment plan and some unfavourable anatomies (mainly oesophagus, PVs, and left bronchus very close together) may not allow it. In our series, one patient was excluded due to the oesophagus located behind the left PVs and in one patient the oesophagus and left bronchus were located so close to the superior PV ostia that the STAR treatment was planned deeper in the veins to avoid their damage. In the last case, the ES after STAR documented a suboptimal PV isolation since the PV antral electrical activity was widely recorded.

Finally, we highlighted mild pericarditis and asymptomatic effusions during follow-up. It is necessary to monitor these events over a long time for potential development of chronic pericarditis and, in addition, to understand a possible correlation with arrhythmic recurrences.

For the ‘torsade de pointes’, it is not possible to determine if this event was due to STAR or to a chance because in the patient’s clinical history there are factors that can be associated with this type of event. In particular, the patient had a syncope about 5 years ago and mild prolonged QT interval on ECG (corrected QTc 480 ms) and was suffering from hypertensive heart disease (ventricular septum diameter 15 mm). His treating physician decided to resume the flecainide therapy 7 days before STAR due to daily symptomatic AF episodes. After 1 h from STAR, the patient had a torsade de pointes treated effectively by electrical cardioversion and he was admitted to the cardiology department. Coronary angiography, echocardiogram, serum electrolytes, and troponin were normal. The patient underwent cardiac ICD implantation. During his follow-up visits, very early ventricular ectopies (minimum coupling interval 290 ms) were documented and the ICD recorded only one atypical atrial flutter after 1 year from STAR. The patient is in good general condition with a significant improvement in the quality of life.

Regarding the secondary endpoints, the efficacy data have several limits (small number of patients, the analysis of recurrences carried out only through ECG and 15 days ECG-Holter monitoring, and the absence of a control group). However, our data report a trend towards a strong reduction in arrhythmic events, a reduction in the intake of AAT, and an improvement in the patients’ quality of life. According to recent data,^22^ atrial arrhythmia-free survival rate after AF ablation are variable, ranging from 70% to 86% in elderly patients (data comparable with our results). This is a pilot study with a limited sample size; further studies are needed to compare efficacy and safety of STAR with catheter ablation or ‘ablate and pace’ strategy in elderly patients.

Three patients developed a form of persistent AF and refused the catheter ablation. These patients had a cardiac valvulopathy that predisposed them to an atrial arrhythmic substrate worsening, even if catheter ablation was performed.^15,19,28^

In terms of ES, we have demonstrated for the first time that STAR is effective in performing PVI even 12 months after treatment. Regarding pathophysiological mechanisms, previous studies reported that myocardial inflammation and structural changes can be induced within a month after STAR, while fibrosis develops after several months.^2^ Later physiological changes are mainly fibrosis, the maturation of which takes several months to complete. The PVI demonstrated 6 and 12 months after STAR is probably the result of a combination of these mechanisms but mainly tissue fibrosis.

In terms of treatment dosimetry, our treatment plan has been performed to minimize the radiation dose to heart and close organs, conforming dose to PVs. However, despite dose to healthy organs (including heart, lungs, bronchus, oesophagus, and all body) respected the radiation dose constrains, as previously reported,^13^ long-term FU will be important to assess possible adverse events due to radiation exposure even for AF and VT. In the absence of data for the latter procedure in arrhythmias, in this study, we enrolled elderly people to reduce the long side effect risk in a population with lower life expectancy respect to younger.

The main limitations of our treatment concern the anatomical characteristics of patients and the absence of a pre-treatment arrhythmic substrate analysis.

Furthermore, due to a non-invasiveness design of the present trial, an electroanatomical map to analyse the arrhythmic substrate was not performed. However, the ablative target in patients with paroxysmal AF is PVI that was achieved with STAR.^15,19^ Elderly patients with paroxysmal AF may have fibrosis located in areas other than PVs, but there are currently no studies demonstrating an antiarrhythmic advantage in ablation of these areas, and, therefore, PVI remains the main ablation target.^19,22^ In the future, studies supported by non-invasive cardiac mapping will allow us to improve this treatment.

## Conclusions

The present phase II trial demonstrated the feasibility of STAR in AF elderly patients, reporting also promising data in terms of safety, outcomes, and quality of life. This new non-invasive and outpatient therapeutic approach can represent a valid alternative for a rapidly growing category of patients.

Close collaboration between radiation oncologists, cardiac electrophysiologists, and medical physics is paramount for this technique, to minimize patient risk and achieve highest levels of ablation accuracy. Further data are needed to confirm our results and to assess possible long- term side effects.

## Data Availability

All relevant data are within the manuscript and its supporting information files.
